# The Correlation of Mineral Density of Jaws With Skeletal Bone and Its Effect on Implant Stability in Osteoporotic Patients: A Review of Patient-Based Studies

**DOI:** 10.7759/cureus.27481

**Published:** 2022-07-30

**Authors:** Sweta G Pisulkar, Rohit A Mistry, Sharayu Nimonkar, Chinmayee Dahihandekar, Gajanan Pisulkar, Vikram Belkhode

**Affiliations:** 1 Prosthodontics, Sharad Pawar Dental College, Datta Meghe Institute of Medical Sciences, Wardha, IND; 2 Department of Orthopaedics, Datta Meghe Institute of Medical Sciences, Wardha, IND

**Keywords:** osseointegration, primary implant stability, implant prosthesis, bone mineral density, s: osteoporosis

## Abstract

Osteoporosis has been an enigma in terms of the administration of implant therapy. It has been implicated as a cause of implant failure as it directly affects the quality of the bone. The diagnosis of osteoporosis is mainly done by measuring skeletal bone mineral density (BMD). During implant therapy, the BMD of jaws can be evaluated on routine orthopantomogram (OPG) or cone beam CT (CBCT). The various advantages of CBCT include establishing a correlation between skeletal bone density and bone density of jaws and estimating its effect on implant stability in osteoporotic patients, which in turn will help in determining the prognosis of the implant in osteoporotic patients. This review is a summary of all patient-related studies conducted in the mentioned context of implant placement in patients with osteoporosis, treatment modalities, and prognosis. We performed a search of relevant articles on Google Scholar, PubMed, and Cochrane, which yielded a total of 25 articles for full-text reviews. After excluding some articles based on the exclusion criteria, a review was conducted along with a pilot study on implant placement in osteoporotic patients. Regional bone density can be a helpful parameter in predicting primary implant stability and it can be a useful indicator of skeletal BMD. With a careful evaluation of BMD, dental implants can be placed in patients with osteoporosis with a better prognosis for the treatment.

## Introduction and background

Branemark in 1965 introduced implant osseointegration, after which prosthetic dentistry took a turn for treatments that produced improved masticatory performance, aesthetics, and comfort for patients with completely or partially missing teeth. Since then, the osseointegrated implant-supported prosthesis has replaced conventional removable prosthodontics treatments. Successful osseointegration depends partly on the condition of the bone and its ability to heal. Bone quality and quantity have been considered important criteria for successful implant therapy. The chances of success of implant therapy are more if the quality and quantity of the bone are more favorable. Bone mineral density (BMD) is one aspect reflecting bone quality. BMD measurements deliver information about achievable implant anchorage and are used to preoperatively estimate primary implant stability or immediate loading capacity. This information should be used to decide upon optimum implant types and to optimize implant positioning and preparation techniques [1,2]. Osteoporosis is a major bone disorder that accounts for about 1.3 million fractures every year in the United States of America. Osteoporosis is characterized by loss of bone mass or skeletal osteopenia, to the level that the bone cannot provide adequate structural support. It has been suggested, but not clearly established, that the osteopenia associated with osteoporosis is also related to bone loss in the mandible. Edentulous men undergoing vestibuloplasty procedures due to severe residual ridge resorption have been reported to have a significantly low bone density in the radius when compared to a control group of healthy age-matched individuals. Successful implant osseointegration has been clearly attributed to good primary stability. It is dependent on the quality and quantity of the bone; primary stability is also related to implant geometry and site preparation [3,4]. Poor primary stability indicates a future failure of the implant due to failure in osseointegration; other reasons for the failure of the implant include inflammation, bone loss, and biomechanical overloading. Poor bone quality is one of the critical areas for primary implant stability, which can compromise the osseointegration process. In the diagnosis of osteoporosis, BMD measured over a large area is considered; on the other hand, for implant therapy, localized BMD is important.

Knowledge about the bone quality in the area of interest is necessary to place the implant, compared to the determination of a representative BMD value averaged over large areas. The knowledge of aesthetics is also important for implant therapy [5,6]. This review investigates the correlation between BMD of mandible and primary implant stability in osteoporotic patients. We believe its findings would contribute to establishing BMD as a prognostic parameter in the success of implant therapy in osteoporotic patients.

## Review

Materials and methods

**Figure 1:**
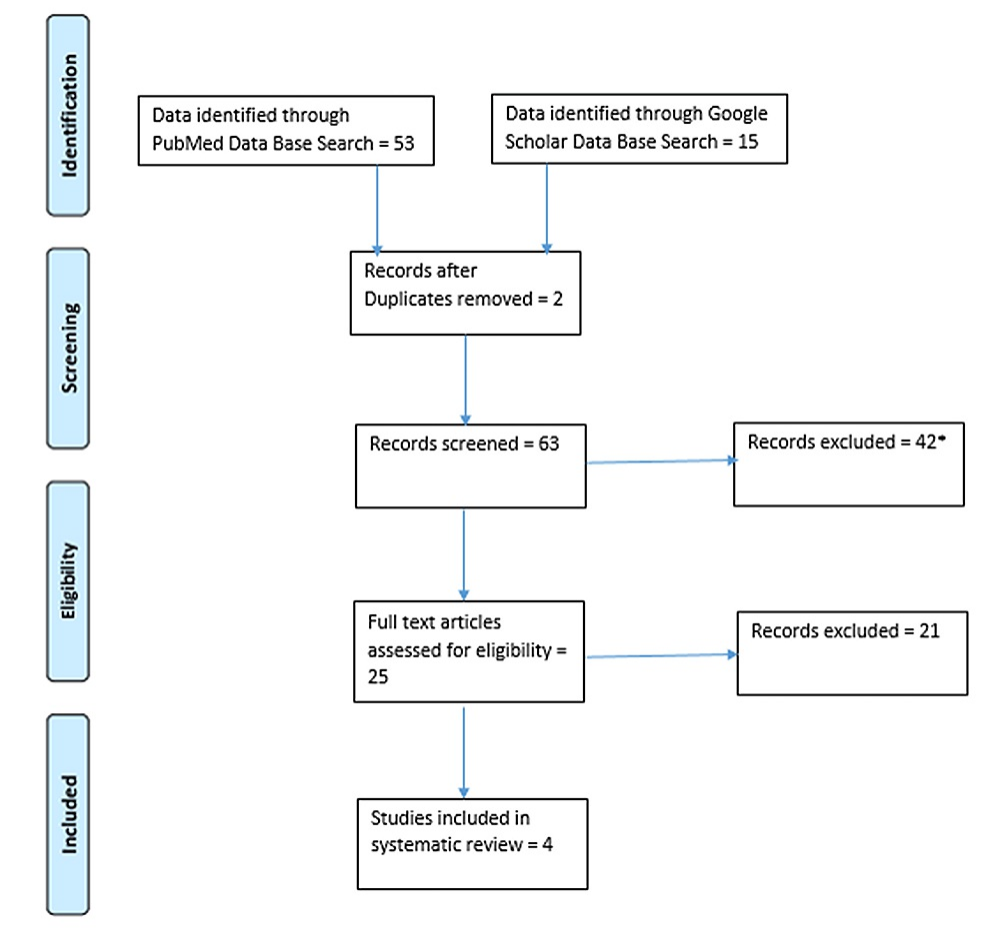
PRISMA flowchart for the studies included in the systematic review *The articles excluded after reading the title PRISMA: Preferred Reporting Items for Systematic Reviews and Meta-Analyses

The protocol for this review was registered with the International Prospective Register of Systematic Reviews (PROSPERO) with the registration number CRD42020193612. We followed the Preferred Reporting Items for Systematic Reviews and Meta-Analyses (PRISMA) guidelines to conduct this review. The PRISMA flowchart for the selected studies is given in Figure 1.

The search strategy is summarized in Table 1.

**Table 1:** Flowchart showing inclusion of articles based on Google Scholar search The keywords used were Bone Mineral Density, Implant Stability Quotient, Edentulous, Osteoporosis with Boolean operators 'AND' and 'OR', and searched on Google Scholar search engine database

Search results combined after screening the Google Scholar database: 15

Articles not in the English language excluded: 1

Articles excluded after reading the title: 1

Duplicate articles excluded: 2

12 articles were searched for full texts, after which 3 articles were excluded, and a total of 9 articles were selected for the review

The assessment was based on the PICO (population, intervention, control, and outcomes) study criteria. The electronic search on the Google Scholar database provided a total of 14 articles that were considered potentially relevant. The texts found using the “[AND] & [OR]” Boolean operators in between the search words Bone Mineral Density, Implant Stability Quotient, Edentulous, and Osteoporosis were 15.

In the second phase of article selection, one article was excluded as it was not found to be in the English language. Another article was excluded after reading the title, and all the duplicate articles were excluded. After reading the full text, a total of 12 articles were selected for the systematic review. Of these 12 articles, three articles were excluded and a total of nine articles were selected for the systematic review.

Discussion

Pathophysiology of osteoporosis and BMD derangement: osteoporosis is characterized by a decrease in BMD and when resorption outweighs apposition. There are many factors that are present by default and make one susceptible to osteoporotic changes. These factors also explain the role they play in decreasing BMD.

As for bone size, increased bone size is proportional to higher BMD. In males, the growth of long bones lasts longer post-puberty when compared to females, giving males an additional advantage of more bone quantity. Osteoporosis will have an effect on females sooner than on males due to this additional growth advantage [7,8].

Regarding age-related bone loss, post menopause, during the first year the bone loss is excessive. It is of greater magnitude in regions with a trabeculae pattern, which leads to initial perforation being followed by total trabecular loss. In males, the mechanism is the same but the magnitude is lower when compared to females [9].

Changes seen after hormonal derangement include a post-menopausal rapid decrease in resorptive agents, like 17β-estradiol secretion, which leads to increased secretion of resorptive osteoclasts. The results lead to an increase in bone resorption. Men with low bioavailable 17 β-estradiol levels have lower BMD, and higher levels of biochemical bone turnover markers (BTM) are associated with accelerated bone loss [10-13].

For patients with secondary hyperparathyroidism, the major cause is the deficit of vitamin D and calcium [4,14], which is a major factor known to cause bone resorption. For patients with hereditary factors, no strong evidence to prove direct heritable components has been observed. Still, a major chunk of cases with bone resorption occurs because of them [4]. For patients whose life is influenced by lifestyle factors like alcoholism, smoking, calcium deficiency, and lack of exercise, the incidence of bone resorption mentioned in the literature is higher. These factors are interrelated [4]. For patients with systemic diseases, thyroid-related disorders like hyperthyroidism, Cushing's syndrome, and other disorders like biliary cirrhosis, hypogonadism, chronic obstructive pulmonary disease (COPD), and beta-thalassemia, the amount of bone resorption witnessed is higher [4,15]. Patients under drug therapy, especially corticosteroids (particularly long-term oral use), selective serotonin reuptake inhibitors (SSRIs), aromatase inhibitors, thiazolidinediones, loop diuretics, proton pump inhibitors, thyroid hormone excess (mainly suppressive treatment after thyroid cancer), anti-androgen treatment (gonadotropin-releasing hormone agonists, surgical castration), and some drugs used in the treatment of AIDS (mainly tenofovir, protease inhibitors), witness higher rates of bone resorption [6]. Correlation between BMD of the skeletal region and BMD of jaws is seen, and the implant success rate is dependent on the quality of bone in which it is to be placed. The BMD that is used to calculate deterioration of the quality of bone in osteoporotic patients is that of larger bones or vertebrae. Dental implants are placed in the maxilla and mandible. This is the rationale behind the importance of knowing the BMD status of the maxilla and mandible. The following studies have correlated skeletal BMD with BMD of jaws (Table 2).

**Table 2:** Flowchart showing inclusion of articles based on PubMed Search The keywords used were Bone Mineral Density, Implant Stability Quotient, Edentulous, Osteoporosis with Boolean operators 'AND' and 'OR', and searched on the Pubmed search engine database

Search results combined after screening the PubMed database: 53

Articles excluded after reading the title: 40

13 articles were searched for full texts, after which 12 articles were excluded, and a total of 1 article was selected for the review

Duplicate articles excluded: 0

In 2007, Drage et al. carried out a study correlating skeletal BMD of the lumbar spine and hip to BMD in the ramus, body, and anterior maxilla [15]. The results showed that BMD in the ramus of the mandible was seen to be equal to the BMD in the femur region but lower than BMD in the lumbar spine region. BMD of the anterior mandible and body was seen to be greater than BMD of the anterior maxilla. The BMD of ramus showed moderately strong relationships with the standard measures of BMD in the spine and hip. In another study by Miliuniene et al. [16], which was conducted in 2008 with the main aim of assessing skeletal BMD of the lumbar spine and jaw BMD cortical thickness of the mandible, the results confirmed that low morphometric parameters are indicative of a higher tendency for osteoporosis.

In a study by Gulsahi et al. in the year 2013, which was performed with the primary aim of assessing skeletal BMD of the femoral neck and jaw BMD of the anterior bicuspid and molar region of the upper and lower jaw, the authors concluded that the mean BMD of maxilla at the molar region is more than the mean BMD of maxilla at premolar and anterior regions. Maxillary and mandibular BMD were not correlated with femoral BMD. Aggarwal et al. in the year 2015 conducted a study with the primary aim of analyzing skeletal BMD of the lumbar vertebra and the jaw BMD of the mandibular cortical index (MCI) [18]. This study concluded that there is a significant correlation between MCI and BMD of the lumbar vertebrae as determined by the dual-energy X-ray absorptiometry (DEXA) [19].

Effect of BMD on implant stability: the success of implant therapy is measured in numerous ways; out of all the possible methods, the measurement of implant stability is a reliable method that helps in deciding whether to load the implant now or later. The implant stability is measured by resonance frequency analysis, which actually determines the success of the osseointegration of implants. Olivé et al. (1990) concluded in a study that implant stability is essential for optimal function [19]. Song et al. (1998) studied the correlation between bone quality and primary implant stability and proved that bone quality needs evaluation before the placement of the implant [20]. Turkyilmaz et al. (2006) assessed and established a strong correlation between bone density and implant stability in the Branemark system (p<0.001); they also emphasized that pre-surgical CT will help in predicting primary implant stability [21]. Javed et al. (2010), in a systematic review, concluded that there is a positive association between implant primary stability and BMD of the receptor site [22].

Considerations for implant therapy in osteoporotic patients: there has been no solid evidence to prove that osteoporosis is a contraindication for implant therapy; however, the decreased bone density due to causes like low estrogen in post-menopausal women can be a source of concern [20]. There is an overall 10.9% failure rate in osteoporotic patients, which is in line with previous studies performed on patients not suffering from osteoporosis [21-23]. The incidences of peri-implantitis are comparable with those of healthy population groups receiving similar therapy [24].

**Figure 2:**
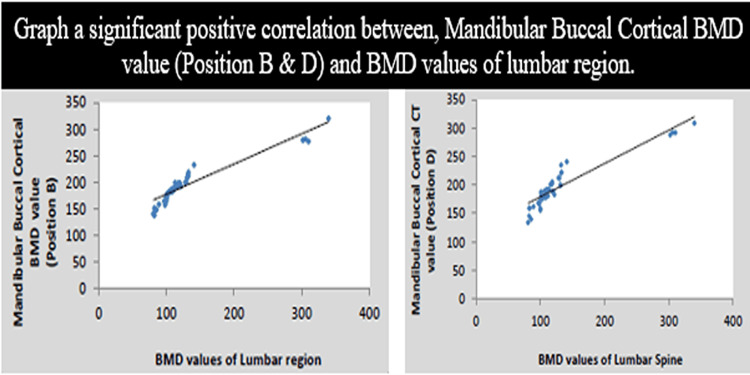
Correlation between mandibular buccal cortical BMD with lumbar BMD: bone mineral density

A study has been carried out in the Department of Prosthodontics, Sharad Pawar Dental College, for which approval from the institutional ethical committee was obtained (DMIMS(DU)/IEC/2016-17/6030). The main aim of the study was to perform a comparative evaluation of CT-derived BMD values of the mandible with that of the lumbar vertebra in elderly edentulous patients and its influence on primary implant stability. A total number of 45 elderly and completely edentulous patients (age group: 0-70 years) who were willing to participate in the study and filled an informed consent were included. Patients with a history of metabolic bone disease were excluded. Two positions were considered on the mandibular jaw bone B & D according to Misch and were correlated with the lumbar vertebra with the help of CT. A graphical representation of the details mentioned in the study is shown in Figure 2.

Statistical analysis was performed using descriptive and inferential statistics using Pearson’s correlation coefficient and one-way ANOVA. The software systems used were SPSS Statistics version 17.0 (IBM, Armonk, NY) and Epi Info version 6.0 (Centers for Disease Control and Prevention, Atlanta, GA). A p-value <0.05 was considered statistically significant. This research demonstrated that the primary implant stability measured using resonance frequency analysis depends and the bone density values and the implant location. Although the prevalence of osteoporosis is on the rise among elderly edentulous patients, the results indicate that primary implant stability [implant stability quotient (ISQ) values] showed a statistically positive significant correlation between BMD values and average maximum torque, justifying the fact that the immediate loading protocol can be indicated even in osteoporotic patients. It was concluded that a correlation of BMD values of the mandible and lumbar vertebra in elderly edentulous patients was established.

It is proven that metabolic bone problems associated with osteoporotic subjects contribute to alveolar bone loss and reduced implant stability [25]. Thus, it is assumed that dental implant placement might be contraindicated in osteoporotic subjects, based on the premise that this pathology may affect the jaw bones similarly [26-29]. Thus, based on the following observations, it can be stated that implant success is dependent on implant stability, which is an indicator of osseointegration; this implant stability has a direct relationship with the regional bone quality. The regional bone quality has a proven correlation with skeletal BMD.

## Conclusions

We believe this review will contribute to establishing that regional bone density can be a helpful parameter in predicting primary implant stability and it can be a useful indicator of skeletal BMD. However, the correlation is weak and needs further research to be proven. Skeletal BMD methods are expensive and not available extensively, whereas regional bone density can even be arbitrarily gauged by easier means such as an orthopantomogram (OPG) or the currently trending method of cone beam CT (CBCT), which is more widely available and is also economically feasible. The onus of diagnosing still lies with the determination of skeletal bone density.
